# The catalytic and the RNA subunits of human telomerase are required to immortalize equid primary fibroblasts

**DOI:** 10.1007/s00412-012-0379-4

**Published:** 2012-07-14

**Authors:** Pamela Vidale, Elisa Magnani, Solomon G. Nergadze, Marco Santagostino, Gael Cristofari, Alexandra Smirnova, Chiara Mondello, Elena Giulotto

**Affiliations:** 1 Dipartimento di Biologia e Biotecnologie “Lazzaro Spallanzani”, Università degli Studi di Pavia, Via Ferrata 1, 27100 Pavia, Italy; 2 Swiss Institute for Experimental Cancer Research (ISREC), School of Life Sciences, Frontiers in Genetics National Center of Competence in Research, Ecole Polytechnique Fédérale de Lausanne (EPFL), 1015 Lausanne, Switzerland; 3 Present Address: Institute for Research on Cancer and Aging, Nice (IRCAN), INSERM U1081, CNRS UMR 7284, Faculty of Medicine, University of Nice—Sophia-Antipolis, F-06107 Nice, France; 4 Istituto di Genetica Molecolare (CNR), Via Abbiategrasso 207, 27100 Pavia, Italy

## Abstract

Many human primary somatic cells can be immortalized by inducing telomerase activity through the exogenous expression of the human telomerase catalytic subunit (hTERT). This approach has been extended to the immortalization of cell lines from several mammals. Here, we show that *hTERT* expression is not sufficient to immortalize primary fibroblasts from three equid species, namely donkey, Burchelli’s zebra and Grevy’s zebra. In vitro analysis of a reconstituted telomerase composed by hTERT and an equid RNA component of telomerase (TERC) revealed a low activity of this enzyme compared to human telomerase, suggesting a low compatibility of equid and human telomerase subunits. This conclusion was also strengthened by comparison of human and equid TERC sequences, which revealed nucleotide differences in key regions for TERC and TERT interaction. We then succeeded in immortalizing equid fibroblasts by expressing hTERT and hTERC concomitantly. Expression of both human telomerase subunits led to telomerase activity and telomere elongation, indicating that human telomerase is compatible with the other equid telomerase subunits and proteins involved in telomere metabolism. The immortalization procedure described herein could be extended to primary cells from other mammals. The availability of immortal cells from endangered species could be particularly useful for obtaining new information on the organization and function of their genomes, which is relevant for their preservation.

## Introduction

Telomeres are peculiar nucleoproteic structures located at the end of linear eukaryotic chromosomes. Vertebrate telomeric DNA is composed of tandem repetitions of the TTAGGG hexamer and is organized in a heterochromatic structure bound to a specific protein complex called shelterin (Palm and de Lange 2008). Telomeres are key components for the maintenance of genome integrity and stability allowing cells to distinguish between the extremities of DNA double-strand breaks and natural chromosome ends, therefore preventing inappropriate DNA repair events (telomere/telomere fusions and telomere/double-strand break fusions) (Xin et al. 2008; O’Sullivan and Karlseder 2010). It was recently demonstrated that telomeres are transcribed by RNA polymerase II in TElomeric Repeat-containing RNA (TERRA) (Azzalin et al. 2007; Schoeftner and Blasco 2008) from promoters located in subtelomeric regions and shared, in humans, among multiple chromosome ends (Nergadze et al. 2009; Farnung et al. 2010). Although the role of this RNA in telomere physiology still needs to be investigated in detail, it has been suggested that TERRA molecules may help in shaping telomere structure and/or act as negative regulator of telomerase activity.

The DNA replication machinery is unable to replicate telomeres completely. Therefore, in the absence of telomere lengthening mechanisms, they progressively shorten during successive cell divisions (Harley et al. 1990). The correct replication of telomeres and, thus, the bypass of the “end replication problem”, is performed by the specialized enzyme telomerase (Collins 2008). The main components of telomerase are a catalytic subunit endowed with reverse transcriptase activity (TERT, TElomerase Reverse Transcriptase) and an RNA moiety containing the template for the synthesis of the telomeric hexamers (TERC, TElomerase RNA Component). Chen et al. (2000) carried out an extensive comparative analysis of TERC from several vertebrate species. They revealed that TERC sequence and structure are remarkably conserved and contain eight major conserved regions (CR 1-8). The pseudoknot domain, located in the 5′ region, is of particular importance since it comprises the template sequence for telomeric repeat synthesis (Chen et al. 2000; Chen and Greider 2003). Mutations in this region reduce or completely abolish telomerase activity (Chen and Greider 2003). Telomere synthesis involves the reverse transcription, catalyzed by TERT, of the telomeric repeat template located in the pseudoknot; it is worth noting that this activity can be reconstituted in vitro in rabbit reticulocytes lysates by co-expressing TERC and TERT (Weinrich et al. 1997; Beattie et al. 1998; Garcia et al. 2007; Collins 2008).

In human tissues, *TERC* is ubiquitously expressed (Feng et al. 1995; Yi et al. 1999), whereas TERT levels are more strictly regulated. In normal somatic cells, *TERT* is expressed at low or undetectable levels (Masutomi et al. 2003), and therefore telomerase activity is not sufficient to maintain telomere length. Thus, telomeres progressively shorten until they reach a critical length that induces an irreversible growth arrest known as replicative senescence (Rodier and Campisi 2011). Conversely, telomerase is active in the germ-line, in stem cells and in about 90 % of human cancers; in these cells, telomere length is maintained averting replicative senescence (Harley 2008).

Several groups successfully obtained an undefined extension of the lifespan of different cell types by exogenous expression of a *TERT* gene. In particular, the ectopic expression of human *TERT* (h*TERT*) allowed the immortalization of many human cell lines (Harley 2002; Belgiovine et al. 2008), as well as of cells from other species, including rabbit and bovine lens epithelial cells (Xiang et al. 2000; Wang et al. 2005), bovine microvascular endothelial cells (Buser et al. 2006), sheep fibroblasts (Cui et al. 2003), canine cell lines (Techangamsuwan et al. 2009), mesenchymal stem cells from rhesus monkey (Gao et al. 2008), and various types of porcine cells (Oh et al. 2007). Although several studies showed that TERT-immortalized cells maintain a normal phenotype, others demonstrated that immortalization could be associated with the appearance of cancer-associated changes and neoplastic transformation (Wang et al. 2000; Harley 2002; Mondello et al. 2003; Serakinci et al. 2004; Zongaro et al. 2005; Belgiovine et al. 2008). Cui et al. (2002) reported the occurrence of telomere shortening and chromosome anomalies probably resulting from chromosome end-to-end fusion in hTERT-immortalized sheep fibroblasts expressing low levels of telomerase.

We show here that the expression of h*TERT* alone is not sufficient to prevent the replicative senescence of fibroblasts from three species of the genus *Equus* (donkey, Grevy’s zebra and Burchelli’s zebra). In contrast, we demonstrate that the overexpression of both the catalytic and the RNA subunits of human telomerase prolongs the proliferative capacity of the same cells, which become immortalized.

## Materials and methods

### Cell cultures

Primary donkey fibroblasts were isolated and established from skin biopsies of a male individual; primary fibroblasts from Burchelli’s zebra were a kind gift from Professor Mariano Rocchi (University of Bari, Italy); primary fibroblasts from Grevy’s zebra were purchased from Coriell Repositories. Cells were cultured in Dulbecco’s modified Eagle’s medium, supplemented with 20 % foetal calf serum, 2 mM glutamine, 2 % non-essential amino acids, 1× penicillin/streptomycin. Cells were maintained at 37 °C in a humidified atmosphere with 5 % CO_2_.

### Plasmid construction

The h*TERT* expression vector pCi-hTERT, containing the human telomerase catalytic subunit cDNA under the control of CMV promoter, and the neomycin resistance gene, was used in a previous study (Mondello et al. 2003).

The h*TERC* expression vector pSSP-hTERC was constructed by inserting a fragment containing h*TERC* in the pSSP plasmid (Salzano et al. 2009), carrying the puromycin resistance gene, under the control of the U1 promoter. A 1,249-bp fragment containing the U1-hTERC cassette was extracted from the plasmid PMD-Banshee (Cristofari and Lingner 2006) by digestion with *Hin*dIII and *Bgl*II (Roche), and then ligated to the pSSP vector digested with the same enzymes. The resulting pSSP-H1-hTERC plasmid was digested with *Eco*RI and *Bgl*II (Roche) to remove the H1-RNA promoter present in the pSSP vector. The linear plasmid was then treated with DNA Polymerase I Large (Klenow) Fragment (Promega) to produce blunt ends and recircularized through standard ligation reaction (T4 DNA Ligase, Fermentas).

To construct the plasmid containing horse *TERC* gene (pCavTERC plasmid), *TERC* sequence was PCR amplified from horse genomic DNA using the primer pair CGGAATTCTAATACGACTCACTATAGGGTGGGGGAGAGTGGGT and CGGGATCCACGTGTTTGAGCCGAGTC, designed on the *Equus caballus* telomerase RNA gene sequence (AF221925) and containing *Eco*RI and *Bam*HI restriction sites at their 5′ end. PCR product was digested with *Eco*RI and *Bam*HI and cloned in the plasmid pUC18 (Fermentas) digested by the same restriction enzymes. The specificity of the insert was confirmed by sequencing with the M13 sequencing primers.

### Transfections

Sequential or concomitant transfection of equid cells with the plasmids containing h*TERT* and h*TERC* were carried out using the Fugene 6 transfection reagent, according to the supplier’s instructions (Roche). Forty-eight hours after transfection, selective agents were added (750 ng/ml puromycin for cells transfected with pCi-hTERT; 750 ng/ml puromycin and 400 μg/ml G418 for cells transfected with both pCi-hTERT and pSSP-hTERC). Resistant clones were isolated, pooled, and cultured at standard conditions; after propagation in 10 cm plates, each division was considered as one population doubling.

### Polymerase chain reaction amplification of cDNAs

Total RNA was extracted using Trizol Reagent (Life Technologies) according to standard protocol. Total RNA was treated twice with 2 U of RQ1 RNase-Free DNase (Promega) for 1 h at 37 °C and then purified again with Trizol to eliminate any DNA contaminations. Total RNA (2.5 μg) was reverse transcribed with 100 pmol of oligo(dT)_18_ primer using RevertAid Premium First Strand cDNA Synthesis Kit (Fermentas) following manufacturer’s recommended protocol. hTERT cDNA was PCR amplified in a 25-μl reaction volume containing 1x Green GoTaq Reaction Buffer (Promega), 0.2 mM of each dNTP, 0.5 U of GoTaq DNA polymerase (Promega), and 20 pmol of primers CGGAAGAGTGTCTGGAGCAA and GGATGAAGCGGAGTCTGGA. After a denaturation step for 2 min at 90 °C, the following amplification cycle was repeated 35 times: 95 °C for 15 s, 61 °C for 30 s, 72 °C for 30 s; final extension was carried out for 5 min at 72 °C.

For the control PRKCI gene, the following primers were used: TGATTGGGATATGATGGAGCA and CATCTGGAGTGAGCTGGACA. After a denaturation step for 2 min at 90 °C, the following amplification cycle was repeated 30 times: 95 °C for 15 s, 61 °C for 30 s, 72 °C for 30 s; final extension was carried out for 5 min at 72 °C.

### Cloning donkey, Grevy’s zebra, and Burchelli’s zebra TERC

One hundred nanograms of donkey, Grevy’s zebra, and Burchelli’s zebra genomic DNA was PCR amplified with the same primers used for the horse TERC (see above). PCR profile was as following: initial denaturation at 95 °C for 2 min; 94 °C for 45 s, 65 °C for 45 s, 72 °C for 45 s, repeated for 35 cycles; final extension was carried out at 72 °C for 5 min. PCR products were cloned and sequenced. Sequences were deposited in GenBank with the following accession numbers: EU486822 (*Equus asinus*), EU486823 (*Equus burchelli*), EU486824 (*Equus grevyi*).

### Comparison of human, mouse, and equid TERC sequences

Sequences of human (*Homo sapiens*, NCBI accession number NR_001566), mouse (*Mus musculus*, NCBI accession number NR_001579) horse (*E. caballus*, NCBI accession number AF221925), donkey, Grevy’s zebra, and Burchelli’s zebra TERC were aligned using the software Multalin (http://multalin.toulouse.inra.fr/multalin/multalin.html). Alignments were refined manually.

### Chromosomes preparation and karyotype analysis

To obtain mitotic cells, cultures were treated with 30 ng/ml of Colcemid (Roche) for 3 h. Cells were harvested, centrifuged, and incubated with 0.075 M KCl at 37 °C for 10 min, then fixed in methanol:acetic acid (3:1) overnight. Fixative was changed two times. For karyotype analysis, the cell suspension was dropped onto glass slides, air dried, and stained with 4 % Giemsa solution in phosphate buffer at 37 °C for 30 min. Chromosomes were analyzed using the optical microscope Leica Leitz Laborlux S.

### Fluorescence in situ hybridization

The telomeric probe, a mixture of 1-20 kb-long synthetic (TTAGGG)_*n*_ fragments, was previously synthesized in our laboratory (Azzalin et al. 1997; Bertoni et al. 1994) and was labelled by nick translation with Cy3-dUTP (Perkin-Elmer). Hybridization to metaphase spreads of primary fibroblasts from the three equid species was carried out as previously described (Nergadze et al. 2006). Post-hybridization washes were performed in low stringency conditions at 37 °C in 4x SSC 25 % formamide, 0.1 % Tween20 in 4× SSC. Chromosomes were counterstained with Hoechst 33258. Digital gray-scale images for Cy3, Cy5, and Hoechst fluorescence signals were acquired with fluorescence microscope (Zeiss Axioplan Fluorescence Microscope) equipped with a CCD camera (Photometrics). Pseudocoloring and merging of images were performed using the IP-Lab software.

### Northern blot

Total RNA from horse, donkey, Grevy’s zebra, and Burchelli’s zebra fibroblasts was extracted using Trizol reagent (Invitrogen) according to the supplier’s instructions. For northern blot, 15 μg of total RNA was run in a 1.2 % formaldehyde agarose gel and transferred onto Hybond-N (Amersham). Northern blots were hybridized with a 343-bp fragment of the human TERC gene labelled with [α-^32^P]-dCTP (Megaprime DNA Labelling System, Amersham Pharmacia Biotech, UK); the fragment was amplified by PCR from the plasmid PMD-Banshee (Invitrogen) using primers CGCTGTTTTTCTCGCTGACTT and GTCCTGGGTGCACGTCCCACAGCTCAG.

### Pulsed field gel electrophoresis and Southern blot

DNA plugs were prepared by standard procedures. Digested genomic DNA fragments were separated in a 1 % FMC Bioproducts GTG agarose gel using a CHEF-DR II Pulsed Field Electrophoresis System (BIO-RAD) and a hexagonal electrode. Electrophoresis was performed at 14 °C in 0.5× TBE at 6 V/cm for 12 h with a switching interval increasing from 0.5 to 2 s. Agarose gel was stained with EtBr (10 mg/ml). The DNA was transferred to Hybond-N (Amersham) and hybridized to the [α-^32^P]-dCTP telomeric probe labelled by random priming (Megaprime DNA Labelling System, Amersham Pharmacia Biotech, UK). Hybridization was performed at 65 °C in 7 % SDS, 0.5 M Na_2_PO_4_ (pH 7), and 2 mM EDTA, and the membrane was washed at 65 °C in 0.1 % SDS, 0.1× SSC for 1 h.

### Telomeric repeat amplification protocol assay

Telomeric activity was measured in primary and transfected cells from donkey, Grevy’s zebra, and Burchelli’s zebra by telomeric repeat amplification protocol (TRAP) assay using the kit “TRAPEZE Telomerase Detection Kit” (Chemicon International) according to supplier’s instructions.

### In vitro reconstitution of telomerase and direct telomerase assay

Horse and human TERC RNAs were in vitro transcribed from *Bam*HI-digested templates (pCavTERC and pUC18hTel2) using the T7 Ribomax kit (Promega). Telomerase was reconstituted in the rabbit reticulocyte lysate as previously described (Cristofari et al. 2007). Direct telomerase assays were performed as in Cristofari and Lingner (2006). Briefly, reactions were carried out for 1 h at 30 °C in 20-μl reactions containing 5 μl of RRL-reconstituted telomerase, 50 mM Tris–HCl (pH 8.0), 50 mM KCl, 1 mM spermidine, 5 mM β-mercaptoethanol, 1 mM MgCl_2_, 0.5 mM dATP, 0.5 mM dTTP, 2 μM dGTP, 20 μCi of [α-^32^P]-dGTP (3,000 Ci/mmol), and 1 μM of telomeric primer (TTAGGG)_3_. Products were phenol-chloroform extracted, ethanol-precipitated, resolved on 12 % polyacrylamide-urea sequencing gels, and analyzed with a PhosphorImager.

## Results

### Transfection of equid fibroblasts with h*TERT*

**Fig. 1:**
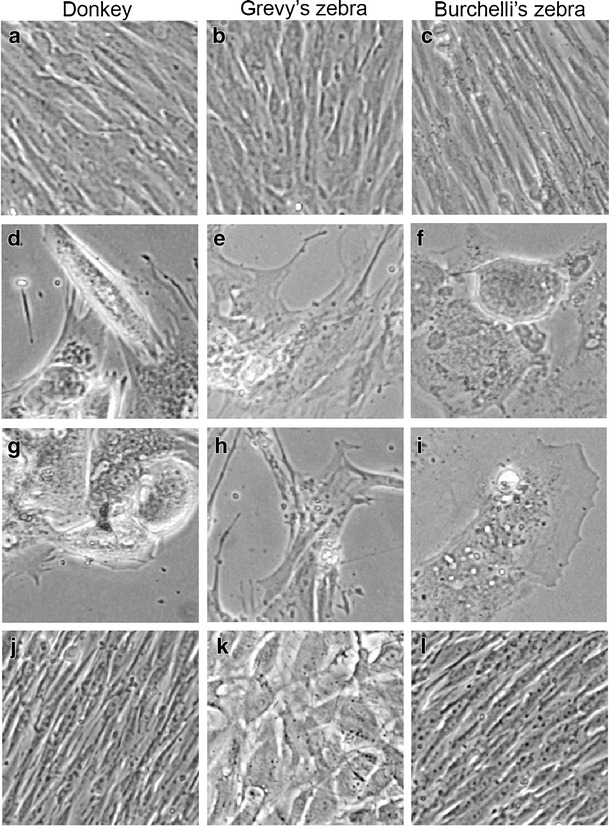
Morphology of donkey (**a**), Grevy’s zebra (**b**), and Burchelli’s zebra (**c**) primary fibroblasts at confluence. Senescent primary fibroblasts from donkey (**d**), Grevy’s zebra (**e**), and Burchelli’s zebra (**f**): cells have an increased size, are round shaped, lose side-by-side organization and vacuolize. h*TERT* transfected fibroblasts from donkey (**g**), Grevy’s zebra (**h**), and Burchelli’s zebra (**i**) showed a limited proliferative capacity, followed by a growth arrest and acquired a morphology similar to senescent primary fibroblasts. Immortalized fibroblasts from donkey (**j**) and Burchelli’s zebra (**l**) are characterized by a normal ordered organization similar to primary cells, while transfected cells from Grevy’s zebra (**k**) show a rounded morphology

A first attempt to immortalize donkey, Grevy’s zebra and Burchelli’s zebra primary fibroblasts was performed by transfecting the cells with a plasmid containing the cDNA for the human catalytic subunit of telomerase and the neomycin resistance gene (pCi-hTERT). At the time of transfection, the donkey, Grevy’s and Burchelli’s zebra fibroblast cell lines had been cultured for 10, 9, and 7 population doublings (PDs), respectively. Cells were actively dividing and showed the typical elongated morphology of primary fibroblasts (Fig. 1a–c). The transfected cells were selected with G418 for 3 weeks, giving rise to a large number (over 200) of resistant clones in all transfections. For each species, two independent pools of clones were propagated.

**Fig. 2:**
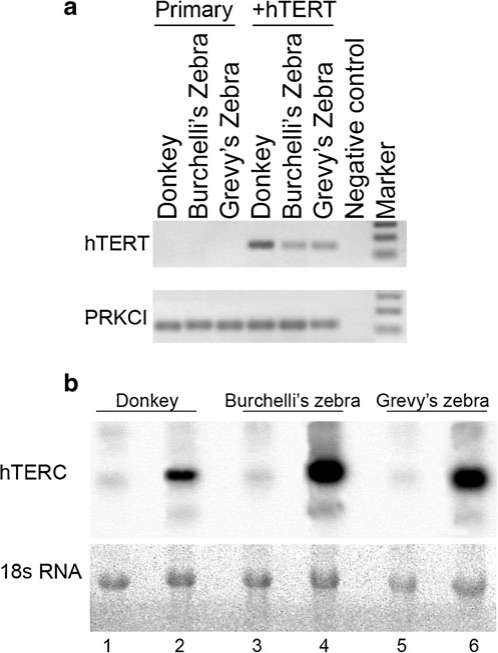
**a** PCR amplification of hTERT and PRKCI cDNA in primary fibroblasts from donkey, Burchelli’s and Grevy’s zebra and cells from the same species transfected with h*TERT* (+hTERT). **b** Top: Northern blot analysis of hTERC expression in primary fibroblasts from donkey (*lane 1*), Burchelli’s (*lane 3*), and Grevy’s zebra (*lane 5*), and in immortalized cells from the same species (lanes 2, 4, and 6). hTERC was detected by hybridization with a ^32^P-labelled fragment of horse TERC. *Bottom*: Ethidium bromide staining of the agarose gel; the band corresponds to the 18S ribosomal RNA

In spite of the successful transfection with the plasmid and of the expression of the h*TERT* gene (Fig. 2a), donkey, Grevy’s zebra and Burchelli’s zebra pools of clones, stopped dividing after about 20, 30, and 10 PDs, respectively, and acquired the typical morphology of senescent cells (Fig. 1 g–i) similar to that of their parental primary fibroblasts at the end of their lifespan (Fig. 1d–f). These results were confirmed in a second independent experiment (data not shown).

Thus, transfection of equid primary fibroblasts with h*TERT* did not lead to cellular immortalization.

## Transfection of equid fibroblasts with h*TERT* and h*TERC*

We hypothesized that the inability of hTERT to immortalize equid fibroblasts could result from limiting TERC levels in equid primary fibroblasts or to an intrinsic incompatibility between human TERT and equid TERC. To overcome these possible limitations, we attempted to produce cell lines expressing both h*TERT* and h*TERC*. To this purpose, we constructed a plasmid containing h*TERC* and the puromycin resistance gene (pSSP-hTERC). We followed two parallel strategies: (1) additional transfection with h*TERC* of the donkey and Grevy’s zebra cells that stably express h*TERT* (sequential protocol); (2) cotransfection of Burchelli’s zebra primary fibroblasts with both h*TERC* and h*TERT* (concomitant protocol). In these two strategies, transfected cells were selected with G418 and puromycin, and polyclonal populations were analyzed.

The donkey or Grevy’s zebra fibroblasts, which were submitted to the sequential protocol, underwent crisis after 15–20 PD. Both populations divided slowly, and most cells acquired a senescent morphology. After crisis, several fast-growing clones appeared; these clones were pooled and expanded. These cells were cultured for over 80 PDs without showing any senescent trait; thus, we considered them as immortal. In contrast, the polyclonal population of Burchelli’s zebra primary fibroblasts cotransfected with both the h*TERC*- and h*TERT*-expressing constructs did not undergo a critical phase, proliferated very fast, and became immortal.

The morphology of donkey and Burchelli’s zebra immortalized fibroblasts was very similar to that of the primary cells, being typically elongated and orderly aligned on the surface of the culture plates (Fig. 1j and l). At the opposite, Grevy’s zebra-transfected fibroblasts were characterized by a round shape and tended to lose the side-by-side organization typical of normal cells (Fig. 1k).

**Fig. 3:**
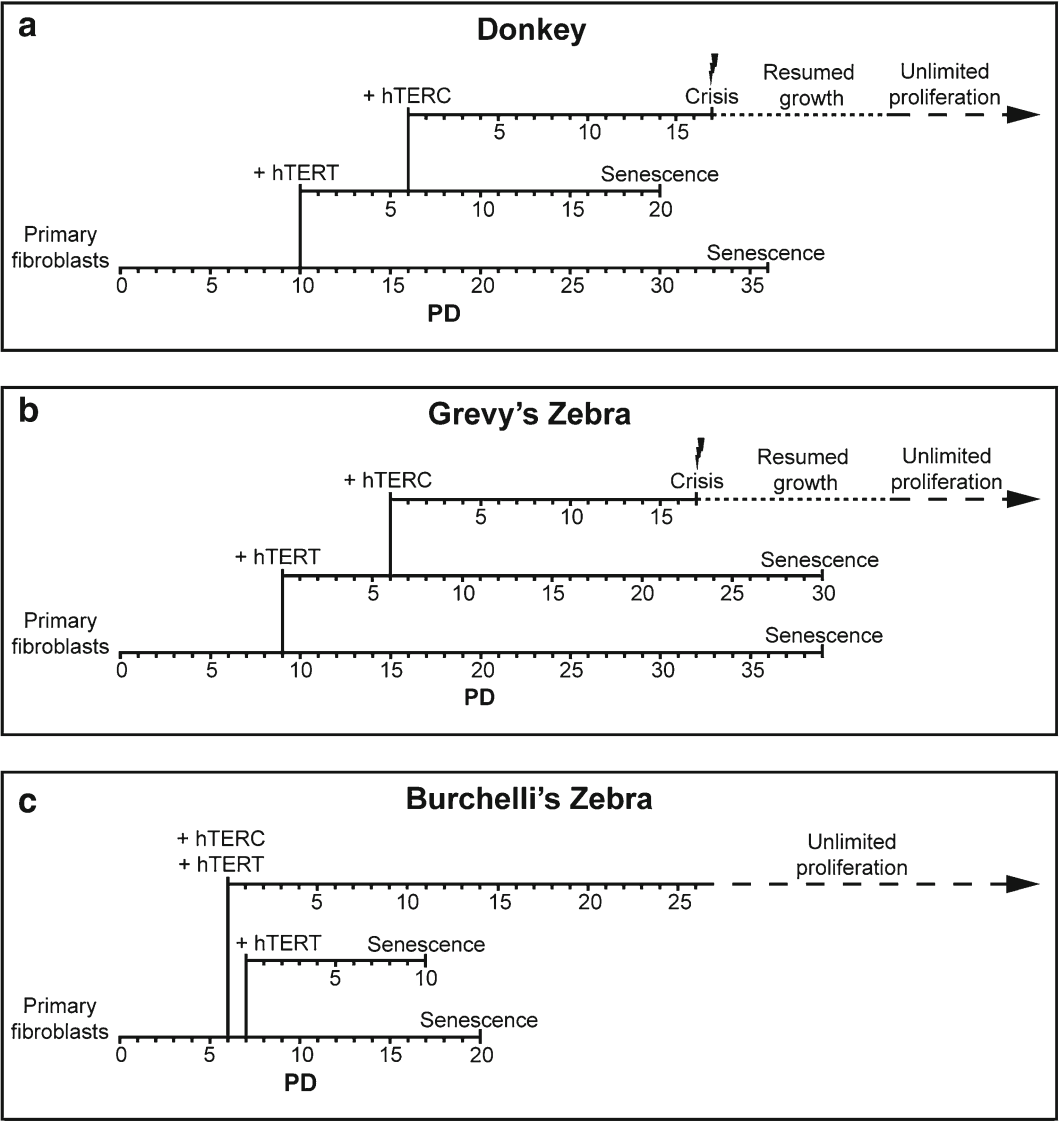
Schematic representation of the immortalization procedures for donkey (**a**), Grevy’s (**b**), and Burchelli’s zebra (**c**) fibroblasts. For each species, the lifespan in population doublings (PDs) of the primary fibroblasts, h*TERT* and h*TERT*/h*TERC* transfected cells is shown. In transfected cells, PDs were re-numbered after transfection

In conclusion, contrary to transfection with h*TERT* alone, transfection with both h*TERT* and h*TERC* can immortalize equid cells. A sketch of the immortalization procedures is depicted in Fig. 3.

## h*TERC* expression and telomerase activity in h*TERT*/h*TERC* transfected equid cells

The expression of the RNA component of telomerase was analyzed by northern blotting using a horse TERC fragment as probe (Fig. 2b). This horse TERC fragment (nucleotides 94–409 in the horse TERC sequence, NCBI accession number AF221925) is identical to the corresponding region in donkey and zebras except for one or two nucleotides, respectively (see Fig. 9, nucleotides 168 and 300), whereas it is 86 % identical to the human TERC fragment.

Using the horse probe, endogenous TERC levels were detected in primary fibroblasts of the three species (Fig. 2b, lanes 1, 3 and 5), indicating that our inability to immortalize cells transfected with h*TERT* alone was not due to a complete absence of TERC expression in these cells. In donkey, Burchelli’s and Grevy’s zebra immortalized cells, strong signals were observed (Fig. 2b, lanes 2, 4, and 6) due to cross-hybridization of the probe with ectopically expressed hTERC.

To test whether telomerase was active in the immortalized cell lines, we performed TRAP assays with total protein extracts from primary cells, h*TERT* transfected cells, and immortalized cells at different PDs. In this assay, the presence of active telomerase in the extract allows the addition of telomeric repeats to an oligonucleotide substrate and the telomerase product is subsequently amplified by PCR leading to a typical ladder.

**Fig. 4:**
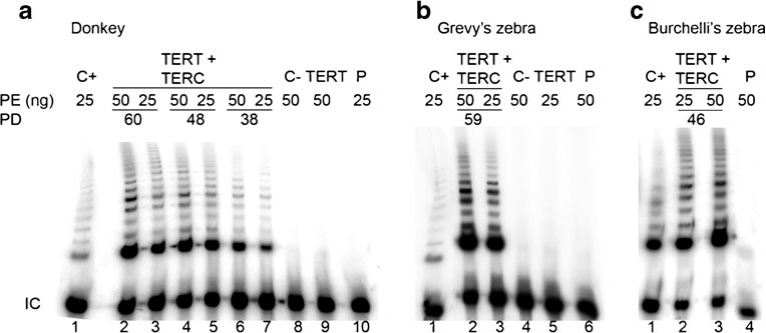
TRAP analysis of telomerase activity in primary fibroblasts (P) from donkey (**a**), Burchelli’s (**b**), and Grevy’s zebra (**c**), and in cells from the same species transfected with h*TERT* only (TERT) or with both h*TERT* and h*TERC* (TERT + TERC). *C+*: positive control prepared using a protein extract from HeLa cells; *C−*: negative control, a heat inactivated protein extract was used in the assay; *IC*: internal amplification control; *PE*: amount of protein extract used for the assay; *PD*: number of population doublings. Telomerase activity was detected in cells transfected with both h*TERT* and h*TERC*, but not in primary fibroblasts or in cells transfected with h*TERT* only

No telomerase activity was detected in protein extracts from primary fibroblasts (Fig. 4a, lane 10; Fig. 4b, lane 6; Fig. 4c, lane 4) and fibroblasts transfected with h*TERT* alone (Fig. 4a, lane 9; Fig. 4b, lane 5). Conversely, fibroblasts immortalized with both telomerase subunits displayed detectable levels of telomerase activity (Fig. 4a, lanes 2-7; Fig. 4b and Fig. 4c, lanes 2-3). Thus, we conclude that human telomerase is efficiently reconstituted in primary equid cells through the ectopic expression of hTERT and hTERC, leading to an active enzyme. These data also confirm that the expression of the human catalytic subunit alone is not sufficient to reconstitute an active telomerase complex in these equid cell lines.

## Telomere elongation in h*TERT*/h*TERC* transfected equid cells

The length of telomeres in primary and immortalized cells of the *Equus* species was estimated by terminal restriction fragment (TRF) analysis. According to this method, genomic DNA was digested with two frequently cutting restriction enzymes, which do not cut within telomeric repeats, resolved by pulsed field gel electrophoresis (PFGE), transferred onto a nylon membrane and hybridized with a telomeric probe. Telomeres appear as a “smear”, which reflects telomere length heterogeneity among chromosomes and among cells.

**Fig. 5:**
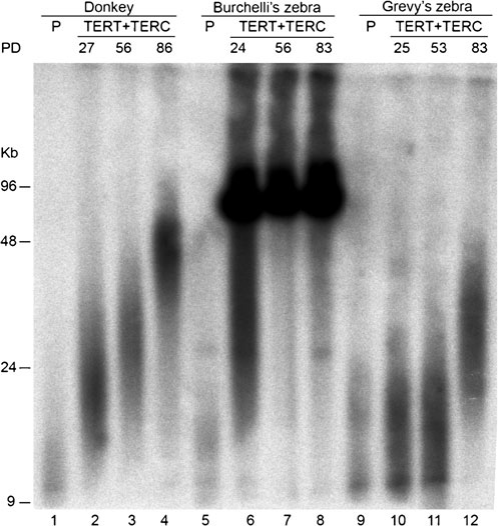
TRF length analysis in primary fibroblasts (P) and cells from donkey, Burchelli’s and Grevy’s zebra transfected with both h*TERT* and h*TERC* (TERT + TERC). *Rsa*I-*Hinf*I digested genomic DNA samples were separated by PFGE and TRFs were visualized by hybridization with a radioactively labeled telomeric probe. TRFs were anayzed at different number of population doublings (PD) for each cell line. In transfected cells, telomeres are clearly elongated compared to primary fibroblasts

Mean TRFs in primary fibroblasts from donkey (Fig. 5, lane 1), Burchelli’s (Fig. 5, lane 5) and Grevy’s (Fig. 5, lane 9) zebra were shorter than 23 kb with a TRF length distribution centered around 9 kb (data from conventional gel electrophoresis, not shown). Cells from the three species exhibit increased telomere length upon cellular immortalization (Fig. 5, compare lanes 4 to 1, 8 to 5 and 12 to 9), although the level of telomere extension was different among the cell lines. Noteworthy, telomeres in Burchelli’s zebra cells that were immortalized by concomitant transfection of h*TERC* and h*TERT* are extremely long (>96 kb), reaching the limit of resolution of the PFGE used here. TRF length was also measured separating the restriction fragments by standard electrophoresis. In this experiment, cells 10 PD after transfection with hTERT alone were also analyzed: although the resolution of high molecular weight fragments was poor, we could conclude that telomere length in primary cells and in hTERT transfected cells was very similar (data not shown).

**Fig. 6:**
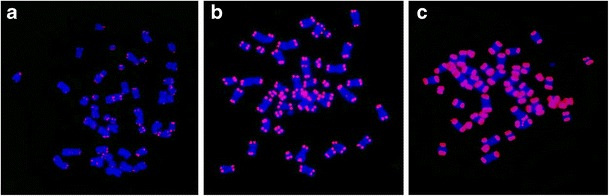
Telomeric fluorescent signal (*red*) variation in metaphase spreads from Burchelli’s zebra primary fibroblasts (**a**) and immortalized cells at PDs 13 (**b**) and PDs 68 (**c**) showing the elongation of telomeres with the increase of PDs. Chromosomes (*blue*) were stained with Hoechst 33258

The presence of extremely long telomeres in the immortalized Burchelli’s zebra cell line was confirmed by cytogenetic analysis. Metaphase spreads from primary fibroblasts and from the immortalized cell line at two different PDs (13 and 68) were hybridized with a fluorescent telomeric probe. All images shown in Fig. 6 were captured using the same exposure time to allow a side-by-side comparison of telomeric signals. In this experimental setting, conditions were chosen to avoid over-exposure of the most intense signals. This prevented the detection of signals on several telomeres of the primary fibroblasts (Fig. 6a). Nevertheless, even in primary cells, all chromosomes showed a telomeric signal when prolonged exposure times were used (data not shown). A clear telomere elongation was detected in immortalized cells at PD 13 (Fig. 6b) compared with primary fibroblasts, and exceptionally intense hybridization signals were observed in the immortalized cells at the last PD at which telomere length was examined (Fig. 6c). The distribution of the signals on the different chromosome ends suggests that all telomeres underwent elongation following ectopic telomerase expression.

In conclusion, both molecular and cytogenetic data demonstrate that the expression of functional human telomerase in equid cells induces immortalization and telomere elongation and that the extent of elongation tends to increase with the number of passages following immortalization.

## Karyotype analysis in h*TERT*/h*TERC* transfected equid cells

**Fig. 7:**
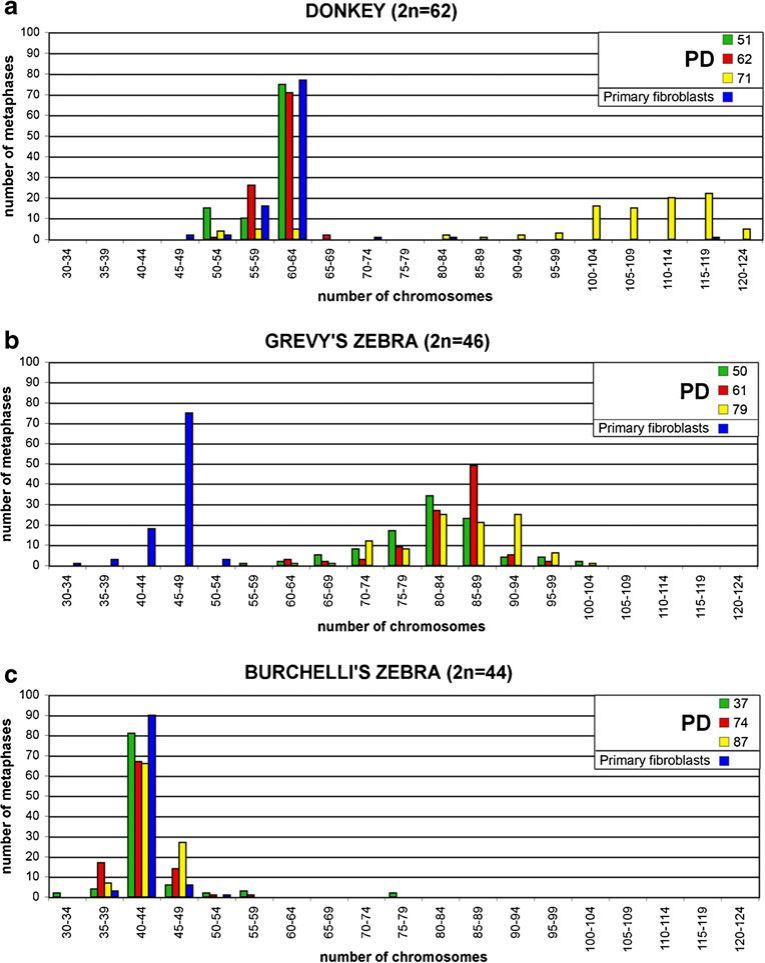
Chromosome number distribution in donkey (**a**), Grevy’s, (**b**) and Burchelli’s zebra (**c**) immortalized fibroblasts at different passages. *Blue bars* represent chromosome number distributions in primary fibroblasts. For each cell type, 100 metaphases were analyzed. The normal diploid number (2*n*) of the three species is indicated. A marked tendency to tetraploidy and subtetraploidy is observed in donkey and Grevy’s zebra immortalized cells

Chromosome spreads were stained with Giemsa and the chromosome number was counted in 100 metaphases of primary and immortalized cells at different passages (Fig. 7). As expected, primary cells showed a modal chromosome number corresponding to the normal diploid complement of each species: 62 for donkey, 46 for Grevy’s zebra, and 44 for Burchelli’s zebra (Fig. 7 blue bars).

Most immortalized donkey cells exhibited a normal number of chromosomes at PD 62, but the majority (90 %) became tetraploid or near tetraploid at PD 71 (Fig. 7a). This suggests that, during prolonged culturing, tetraploid cells endowed with a selective advantage may become prevalent in the population. All the Grevy’s zebra immortalized cells (Fig. 7b) had abnormal karyotypes, with a number of chromosomes varying between 70 and 95. In striking contrast, the majority of immortalized Burchelli’s zebra cells maintained a diploid karyotype (Fig. 7c).

## In vitro analysis of the activity of the telomerase core enzyme reconstituted by combining human TERT and horse TERC

**Fig. 8:**
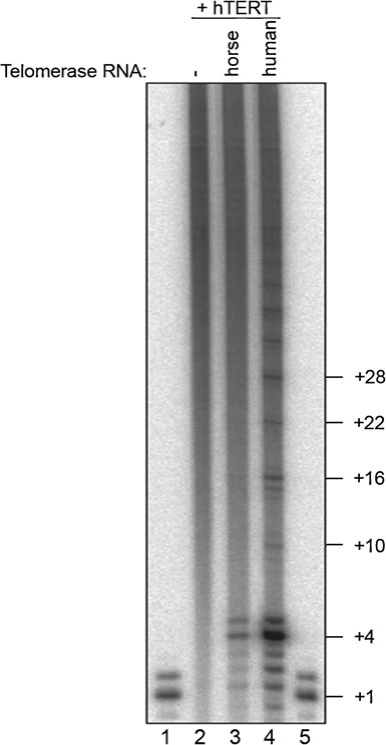
In vitro reconstitution of telomerase with human TERT and horse TERC in the rabbit reticulocyte lysate (RRL) system. Telomerase activity was assayed using telomerase reconstituted without telomerase RNA (lane 2) or with horse (lane 3) or human (lane 4) TERC. *Lanes 1* and *5* contain a size marker, corresponding to the primer radiolabeled at the 3′ end with [α-^32^P]-dGTP. The number of nucleotides added by telomerase is indicated on the right. The total telomerase activity of the chimeric telomerase reconstituted with horse TERC and hTERT is 10 % relative to human telomerase and extension aborts after the first telomeric repeat addition (+4 product)

**Fig. 9:**
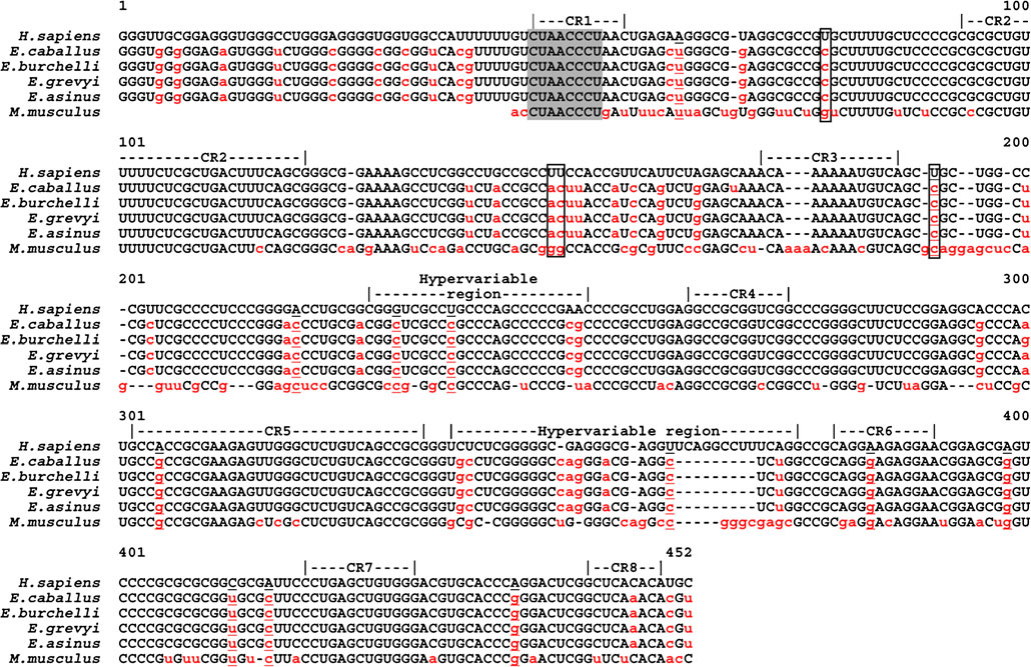
Alignment of human (*H. sapiens*), horse (*E. caballus*), Burchelli’s zebra (*E. burchelli*), Grevy’s zebra (*E. grevyi*), donkey (*E. asinus*), and mouse (*M. musculus*) telomerase RNA sequences. Nucleotides conserved among the six species are in *upper*-*case* and mismatches are in *red lower*-*case*; nucleotides shared between the mouse and the equine sequences, but divergent from the human ones, are *underlined*; nucleotides on *gray background* correspond to the telomeric template; nucleotides relevant for telomerase activity are *boxed* (Chen and Greider 2003); conserved regions (CR1-8) as well as the two hypervariable regions (Chen et al. 2000) are shown

To test whether the inability to immortalize equid fibroblasts by the ectopic expression of hTERT alone was due to an incompatibility between human TERT and equine TERC, we reconstituted the telomerase core enzyme in vitro in the rabbit reticulocyte lysate (RRL) system. We analyzed telomerase activity and processivity by a direct telomerase assay, in which a telomeric primer is extended by telomerase in the presence of [α-^32^P]-dGTP (Cristofari and Lingner 2006). In these experimental conditions, successful reconstitution of active human telomerase by combining the hTERT protein and the hTERC RNA results in the processive extension of the telomeric primer as visualized by a typical 6-nucleotide ladder in sequencing gels (see Fig. 8, lane 4). Interestingly, when human TERC is replaced by horse TERC, an enzyme with reduced—but detectable—activity is assembled, but extension aborts after initial extension of the primer, when the end of the template is reached, suggesting that this chimeric telomerase is impaired in the translocation step of the telomerase reaction, required for repeat addition processivity. Noteworthy, these short abortive products are not detected by the PCR-based TRAP assay, which requires the addition of at least three telomeric repeats to be amplified, in agreement with the results shown in Fig. 4. Altogether, these results suggest an incompatibility between human TERT and horse TERC, which globally reduces telomerase enzymatic activity and abolishes its repeat addition processivity. In donkey and Grevy’s zebra, the sequence of TERC is identical to the horse sequence, except for one nucleotide (Fig. 9, 268U>C), whereas in Burchelli’s zebra, an additional nucleotide is different (300A>G), and these divergent positions are located in functionally irrelevant regions. The hypothesis of incompatibility between human TERT and horse TERC can thus be extended to the other three species.

## Sequence comparison of human, equid, and mouse TERC

We wondered whether differences in the sequence of human and equid *TERC* could explain our observation that the combination of human TERT and horse TERC cannot fully restore telomerase activity.

The human and the horse *TERC* sequences were already available (*Homo sapiens*, NCBI accession number NR_001566; *E. caballus*, NCBI accession number AF221925). We cloned and sequenced the *TERC* genes from donkey, Grevy’s zebra, and Burchelli’s zebra. Sequence alignment of the TERC RNA from these species is shown in Fig. 9. The mouse sequence (*M. musculus*, NCBI accession number NR_001579) was included in the comparison because it was previously shown that, similarly to equine TERC, mouse TERC is unable to restore telomerase activity in combination with human TERT (Beattie et al. 1998; Chen and Greider 2003).

In a previous study, Chen et al. (2000) compared TERC RNA sequences from 32 vertebrates (including human, horse, and mouse) and revealed the presence of eight conserved regions (Fig. 9, CR1–CR8) possessing more than 90 % identity in these species. CR1 includes the template for telomeric DNA synthesis (CUAACCCU). Two hypervariable regions were also detected: the first one is located between CR3 and CR4 and the second one between CR5 and CR6.

The TERC sequence from the equid species is more similar to the human sequence than to the mouse one; in particular, both equid and human TERC contain 44 nucleotides in the 5′ region preceding the template that are missing in mouse TERC. In addition, while 193 nucleotides are divergent between the equid and the murine sequence, the number of diverging nucleotides between the equid and the human sequence is only 53 (54 in the horse; Fig. 9, red nucleotides). In spite of the relative similarity between human and equine TERC, several key positions relevant for TERC/TERT binding are shared between the mouse and the equid TERC sequences. A detailed analysis of these positions and of their possible role in determining TERC/TERT incompatibility is reported in the discussion.

## Discussion

The goal of this work was to develop an efficient system to immortalize cells from *Equus* species and in general from any mammalian species even in the absence of previous knowledge on telomerase gene sequences and enzymatic activity.

We initially attempted to immortalize donkey, Grevy’s zebra, and Burchelli’s zebra primary fibroblasts by ectopic h*TERT* expression. Since h*TERT* expression was not sufficient to induce either telomerase activity or cellular immortalization, we sequentially (donkey and Grevy’s zebra) or simultaneously (Burchelli’s zebra) introduced h*TERT* and h*TERC* in these cells.

Following transfection with h*TERC*, the majority of donkey and Grevy’s zebra cells became senescent and underwent crisis; however, a few fast-growing clones that appeared after crisis gave rise to an immortalized cell population. On the contrary, Burchelli’s zebra fibroblasts, simultaneously transfected with h*TERT*- and h*TERC*-expressing constructs, did not undergo crisis and maintained a high proliferation rate during all the culture period.

In all the immortal populations, we detected high levels of telomerase activity and found that telomere length tended to increase with increasing passages in culture. Telomeres reached lengths much greater than in parental cells, from several tens of kilobytes in donkey and Grevy’s zebra cells up to more than 100 kb in Burchelli’s zebra cells. The elevated telomere lengthening in these cell lines could be due to the overexpression of both hTERT and hTERC, which has been shown to substantially increase telomerase activity in many different cell lines and in human primary fibroblasts compared to the overexpression of a single telomerase subunit (Cristofari and Lingner 2006). The restoration of telomerase activity and telomere lengthening by hTERT and hTERC indicates that all the other equid telomerase subunits and telomeric proteins are compatible with the human telomerase components, ensuring telomerase activity and telomere maintenance in the immortalized cell lines. In the three species, the different degree of telomere elongation did not seem to be related to clear-cut differences in telomerase activity; it could be hypothesized that it reflected variations in telomere chromatin organization leading to a different accessibility of telomeres to telomerase.

Donkey immortalized cells maintained the normal morphology of primary fibroblasts, being elongated and regularly aligned. Nevertheless, these cells showed a tendency to become tetraploid after numerous passages in culture. Grevy’s zebra immortalized cells became tetraploid at earlier passages compared to donkey cells and showed a morphology that was different from that of primary fibroblasts: they were larger, round shaped, and tended to lose the side-by-side organization of primary cells. These karyotype and morphology changes could be indicative of a transition towards a transformed phenotype during culture propagation, as it has been described in different h*TERT*-immortalized human fibroblasts (Mondello et al. 2003; Zongaro et al. 2005; Belgiovine et al. 2008). Burchelli’s zebra immortalized cells preserved a morphology similar to primary cells and maintained the normal diploid number in the majority of the cells. Even though a few aneuploid cells were observed at the latest passages (PD 74 and 87), Burchelli’s zebra fibroblasts immortalized by the concomitant expression of h*TERT* and h*TERC* kept the phenotype most similar to normal cells. The peculiar characteristics of Burchelli’s zebra immortalized fibroblasts may be due to the simultaneous transfection method used, to the genetic background, or to the culturing history of the primary cell line.

We found that ectopic expression of h*TERT* is not sufficient to immortalize equid primary cells. In addition, we observed that the human telomerase catalytic subunit could assemble with the horse telomerase RNA subunit in vitro, but the resulting chimeric telomerase has lost the ability to add multiple repeats to a telomeric DNA substrate. This indicates that hTERT and horse TERC are incompatible and suggests that ectopic hTERT is not able to form an active telomerase complex with endogenous horse TERC in equid cells, preventing telomere extension and cellular immortalization. Our sequence comparison of human, equid, and mouse TERC showed that, although the equid TERC is more similar to the human gene than to the mouse one, some significant differences between human and equid TERC may cause the incompatibility between hTERT and equid TERC. In particular, it is likely that the differences at nucleotides 77, 146, 147, and 184 (boxed in Fig. 9) are relevant in determining the incompatibility between horse TERC and human TERT; in fact, in a previous study Chen and Greider (2003) demonstrated that mutations in the regions including nucleotides 77, 146, and 147 impair binding with TERT, while those in the region containing nucleotide 184 cause a reduction in telomerase activity. Notably, the same nucleotides (77, 146, 147, and 184) are divergent from the human sequence also in the mouse. Another feature shared by the mouse and the equid sequence is a deletion in the second hypervariable region corresponding to the human region comprised between nt 327 and nt 363; although this region does not seem directly involved in the interaction between the RNA and the protein telomerase subunits, it is possible that alterations in the stem-loop structure assumed by this region may influence the enzymatic activity. Finally, it is worth pointing out that at 24 positions, both the mouse and the equid sequences are divergent from the human sequence, and at 12 of these positions, the mouse and the equid sequences share the same nucleotide (nt 62, 184, 210, 221, 227, 295, 349, 371, 386, 401, 405, and 432 in the human sequence; Fig. 9, underlined nucleotides). This situation is reminiscent of the previous observations showing that mouse TERC cannot form an active complex with hTERT (Beattie et al. 1998; Chen and Greider 2003) and that hTERT overexpression in mouse cells inhibits their endogenous telomerase activity (Boklan et al. 2002).

The method described here, that is the cotransfection of primary cells with both human *TERT* and *TERC*, may be particularly useful to obtain stable cell lines from rare individuals of endangered species. Conservation biologists and wildlife managers increasingly rely on DNA analysis tools to identify species, determine sex, and analyze pedigrees; genomics is changing the way we think about conservation (Wolinsky 2012; Seabury et al. 2011). These issues were recently discussed in a dedicated international meeting (Cruz et al. 2012).The availability of easily manageable immortal cells will thus facilitate genomic and post-genomic studies and their applications to address evolutionary, ecological, and conservation questions.

## Concluding remarks

Primary cells have limited proliferative capacity and can be difficult to obtain; therefore, the availability of immortal cells to perform genetic and molecular studies is extremely useful. In this paper, we show that co-expression of human TERT and TERC in different species of the genus *Equus* allows cellular immortalization; in particular, we found that the concomitant expression of both human telomerase subunits gives rise to cell lines that maintain a normal phenotype for several generations and are thus particularly suitable for molecular and cellular studies. It is worth mentioning that we recently discovered that the satellite-less equid centromeres are an ideal model system to study the centromeric function at the molecular level (Piras et al. 2009; Wade et al. 2009; Piras et al. 2010). The availability of immortalized fast-growing cells will greatly facilitate these studies. The immortalization approach that we have set up could be extended to cells from various mammalian species, even in the absence of a cloned *TERT* gene. In particular, it could be used for endangered species for which the availability and/or sequence data might be limiting and the possibility to study immortal cell lines could give new information on genome organization and function relevant for their preservation.
